# Use of workplace foodservices is associated with reduced meal skipping in Korean adult workers: A nationwide cross-sectional study

**DOI:** 10.1371/journal.pone.0243160

**Published:** 2020-12-03

**Authors:** Woo-young Shin, Jung-ha Kim

**Affiliations:** Department of Family Medicine, Chung-Ang University Medical Center, Chung-Ang University College of Medicine, Seoul, Republic of Korea; Yenepoya Medical College, Yenepoya University, INDIA

## Abstract

Skipping meals is a poor eating behaviour known to result in poor diet quality and health outcomes. Nevertheless, it has become increasingly common over the past few decades in many countries. This study aimed to examine the potential association between the use of workplace foodservices and skipping meals among Korean adult workers using data from the Korea National Health and Nutrition Examination Survey 2016–2018, a nationwide cross-sectional survey of a representative Korean population. A total of 5,137 workers aged 20–64 years were included. Dietary assessment was conducted using a 24-hour dietary recall. In total, 41.2% participants skipped one meal or more per day. The percentage of workers who skipped meals was 43.1±1.0% among participants who did not use workplace foodservices compared to 31.8±1.9% among those who did (P<0.01). Skipping meals was significantly associated with not using workplace foodservices, after adjusting for other confounders including sociodemographic variables, health-related variables, and meal procurement source (odds ratio = 3.4; 95% confidence interval = 2.6–4.4; P<0.01). We found a significant association between using workplace foodservices and reduced skipping meals in Korean adult workers. This study suggests the importance of the provision of workplace foodservices for workers to potentially reduce poor eating behaviours such as skipping meals.

## Introduction

Skipping meals is a poor eating behaviour known to result in poor diet quality and health outcomes. Nevertheless, it has become more common over a past few decades in many countries [1,2]. A recent national survey in Korea reported that one in four individuals skipped breakfast, with this behaviour showing steadily increasing rates [3]. Furthermore, it was shown that young adults more frequently skipped regular meals than older people. In Korea, the estimated prevalence rates of skipping meals were 46.5% and 40.1% in male and female adults aged 19–29 years, respectively, and 31.3% and 25.1% in male and female adults aged 30–49 years, respectively [3].

Skipping meals has been assessed in terms of its long-term health effects. It is associated with a lower intake of required nutrients, decreases in cognitive function, and increased risks of adverse health outcomes such as obesity, insulin resistance, and other various cardiometabolic disorders [4–7]. Moreover, it has been shown that eating regularly without skipping meals is associated with potential benefits, including increases in the rates of adequate nutrient intake, leading to improved nutritional quality and healthy dietary habits [8,9]. As such, this dietary behaviour is an important factor for nutrition and health, as it not only helps in preventing and managing diet-related chronic diseases but also contributes to improving the quality of life, work efficiency, and productivity in economic development [10,11].

Despite the significant health impacts of skipping meals and its high prevalence among young adults, studies on the factors associated with this poor eating behaviour are limited. The available evidence suggests that physical or social environmental factors, including housing type, family support, and household socioeconomic status, as well as individual factors, such as a sedentary lifestyle, are associated with skipping meals [12–14]. A study by Rosenrauch et al. found that perceived peer support with regard to healthy eating was associated with skipping meals in adolescents [15]. However, the association of skipping meals with the use of workplace foodservices, which is another potential environmental factor, has never been studied in workers, although the workplace is where most workers spend a major part of the day with their colleagues, and the workplace may directly or indirectly affect their dietary behaviours [16].

Therefore, this study aimed to compare the sociodemographic and health-related characteristics of workers according to whether they skip meals and to identify whether there is an association between using workplace foodservices and reduced meal skipping among Korean workers aged 20–64 years.

## Materials and methods

### Data source and study participants

The Korea National Health and Nutrition Examination Survey (KNHANES) is a nationwide cross-sectional survey that combines interviews with physical and laboratory examinations to assess the health and nutritional status of a representative population in Korea. The survey was conducted by the Korea Centers for Disease Control and Prevention [17]. This study was based on data acquired from the KNHANES VII (2016–2018).

Participants were workers who completed health interviews, physical examinations, laboratory measurements, and dietary surveys. Individuals aged <20 years or >64 years; individuals with a history of cancer, chronic kidney disease, or thyroid disease; patients treated for tuberculosis; and pregnant women were excluded due to the potential of unexpected dietary patterns. Individuals who reported implausible total energy intakes (<500 kcal/d or >4,000 kcal/d) or those who were on a diet were also excluded considering the reliability of the analysis. Written informed consent was obtained from all participants. The KNHANES was approved by the institutional review board of the Korea Centers for Disease Control and Prevention (2018-01-03-P-A).

### Dietary assessment

The dietary survey was conducted using a 24-hour dietary recall method by professional interviewers (two dietitians who were thoroughly acquainted with the protocols and techniques involved in the survey). Data on all foods consumed by the participants for 24 hours during the day prior to the dietary survey were collected [18]. Participants were assigned to the skipping meals group based on whether the participants had skipped at least one regular meal (breakfast, lunch, or dinner) on the day before the survey; this was assessed via “yes or no” questions. Each meal consumed by the participants was categorised into one of three groups: workplace foodservice, eating-out, or homemade (home-cooked) meals. We classified participants into the weekday or weekend group based on the day of the dietary survey.

### Sociodemographic and health-related covariates

Information on the participants’ sociodemographic characteristics, including age, sex, household income, marital status, educational level, and occupation, was gathered through face-to-face interviews or self-reported questionnaires. A married person was regarded as having a married status and as cohabiting. A high education level was defined as having at least college education. Occupations were classified into three groups: non-manual (general managers, professionals, or office workers), service or sales, and manual (skilled agricultural workers, forestry and fishery workers, craft and related trade workers, plant and machine operators or assemblers, or elementary workers).

The health-related behaviours evaluated in this study included cigarette smoking, alcohol consumption, and physical activity. Participants who smoked at the time of the survey or had smoked ≥100 cigarettes in their lifetime were regarded as current smokers. Male and female participants were considered as heavy drinkers if they consumed ≥7 and ≥5 drinks, respectively, at least twice a week. The adequate physical activity group consisted of those who engaged in moderately intense physical activities for at least 150 minutes during the week, vigorously intense activities for at least 75 minutes during the week, or an equivalent combination of moderately and vigorously intense activities [19,20].

Health examinations were conducted by trained personnel according to standardised protocols. Body weight and height were measured while participants were dressed in minimal clothing and without shoes. Each participant was weighted using a digital weight scale (GL-6000-20, G-Tech, Uijeongbu, South Korea). The body weight measurement was captured when the readout on the digital measurement device became stable after the participant stood in the centre of the scale platform with their hands on the sides while looking straight. Height was measured using a stadiometer (seca225, seca^®^, Hamburg, Germany) with a fixed vertical backboard and an adjustable head piece. For the measurement of height, each participant stood up straight against the backboard with the body weight evenly distributed and the back of the head, shoulder blades, buttocks, and heels touching the backboard. The result was captured while the participant was correctly positioned and holding a deep breath [17,18]. Body mass index (BMI) was calculated as the body weight in kilograms divided by the square of height in metres (kg/m^2^). BMI ≤18.5 kg/m^2^ and BMI ≥25 kg/m^2^ were defined as underweight and obesity, respectively. Blood pressure was measured on each participant’s right arm after a 5-minute rest using a standard sphygmomanometer (Wall Unit 33(0850), Baumanometer^®^, NY, US). Venous blood samples were obtained for blood glucose and lipid profile measurements after fasting for 12 hours. In this study, chronic medical conditions were defined according to the data from the health examination, laboratory examination, or self-reported medication history of the participants. Each chronic disease was defined according to the following criteria: hypertension as increased blood pressure (systolic ≥140 mmHg or diastolic ≥90 mmHg) or use of antihypertensive medications; diabetes as a fasting blood glucose level ≥126 mg/dL, self-reported medical diagnosis by a health professional, or use of antidiabetic medications; and dyslipidaemia as a total cholesterol level ≥240 mg/dL, serum triglyceride level ≥200 mg/dL in fasting blood samples, or use of medications for dyslipidaemia.

### Statistical analysis

Statistical analyses were conducted using SAS, Version 9.4 (SAS Institute Inc., Cary, NC, USA). All analyses used sample weights assigned to participants to represent the Korean population, which were determined using a multistage clustered and stratified randomised sampling method based on the national census data [7]. The participants were categorised into two groups according to whether they skipped meals, and characteristics were compared between the groups. Skipping meals was regarded as skipping at least one regular meal per day. Data are presented as means for continuous variables and as weighted percentages for categorical variables (with their standard errors). The differences in sociodemographic and health-related characteristics between participants who skipped meals and those who did not skip meals were assessed using weighted t-tests or χ^2^ tests.

Multivariate logistic regression analyses were used to estimate the association between using workplace foodservices and skipping meals, after adjusting for confounders including age; sex; household income; marital status; educational level; occupation; smoking status; heavy alcohol consumption; adequate physical activity; obesity; chronic diseases including hypertension, diabetes, and dyslipidaemia; meal procurement source; and day of the survey (weekday/weekend). Adjusted odds ratios (ORs) and 95% confidence intervals (CIs) for skipping meals were calculated according to the use of workplace foodservices. For these analyses, P-values <0.05 were considered statistically significant.

## Results

**Fig 1:**
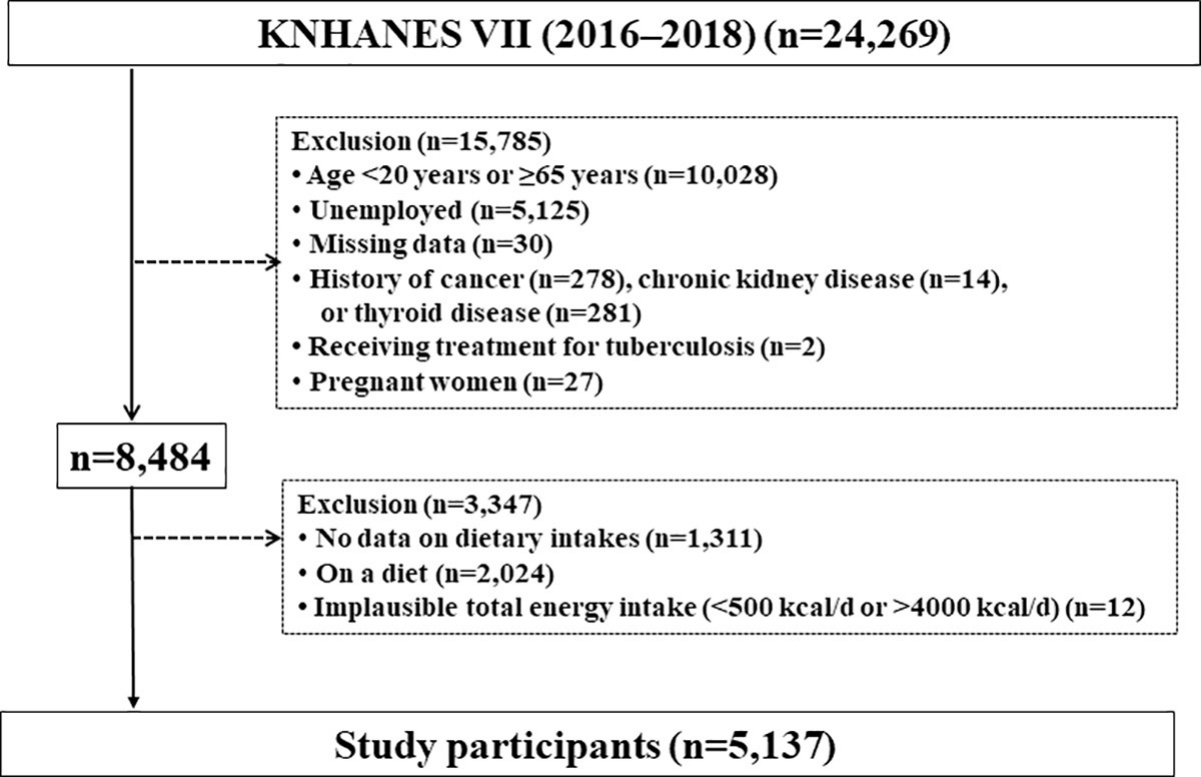
Flowchart for the inclusion of study participants (KNHANES VII 2016–2018).

A total of 24,269 (8,150, 8,127, and 7,992 in 2016, 2017, and 2018, respectively) candidates had completed a health interview/examination and nutritional survey among the Korean men and women selected using a two-stage stratified cluster and complex sampling method in the KNHANES VII (2016–2018). Finally, 5,137 workers were included in the statistical analysis (Fig 1).

### Baseline characteristics of the study participants

Of the 5,137 participants at baseline, more than half of the participants were males (60.3%). The mean patient age was 44.7 years. In total, 41.2% participants skipped at least one meal a day. The baseline characteristics of the study participants stratified according to whether they skipped meals are shown in Table 1. Compared with workers who consumed all three meals a day, those who skipped at least one meal a day were younger; non-manual, service, and sales workers; had lower household incomes; were unmarried; were underweight or had obesity; and were current smokers and heavy drinkers (all P<0.05). In addition, the prevalence of hypertension was lower among workers who skipped at least one meal a day than among those who consumed all three meals a day (P<0.05). All other variables were not significantly different between the two groups.

**Table 1:** Baseline characteristics of study participants according to meal-skipping behaviour.

Variables^b^	Total (n = 5,137)		Skipping meals				P-value^a^
			No (n = 3,206)		Yes (n = 1,931)		
	%	SE	%	SE	%	SE	
Number			58.8	0.9	41.2	0.9	
Age, years	44.7	0.2	47.0	0.2	40.8	0.3	<0.01
Male	60.3	0.7	60.0	0.9	60.6	1.2	0.71
Household income							<0.01
Quartile 1	6.4	0.5	5.9	0.5	7.2	0.7	
Quartile 2	22.3	0.8	21.3	1.0	23.7	1.2	
Quartile 3	32.9	0.9	31.6	1.1	34.9	1.2	
Quartile 4	38.3	1.1	41.2	1.3	34.3	1.4	
Higher education: at least college	51.2	1.1	51.6	1.3	50.6	1.5	0.58
Occupation							<0.01
Non-manual worker	45.1	1.0	44.9	1.2	45.5	1.6	
Service and sales worker	21.4	0.7	18.9	0.8	25.0	1.1	
Manual worker	33.5	0.9	36.3	1.1	29.5	1.4	
Married	68.8	1.0	76.9	1.0	57.1	1.5	<0.01
Obesity							0.04
Underweight	4.6	0.3	3.9	0.4	5.5	0.6	
Obesity	33.4	0.8	33.0	1.0	34.0	1.3	
Current smoker^c^	28.0	0.8	23.7	0.9	34.0	1.3	<0.01
Heavy drinker^d^	17.3	0.7	14.3	0.7	21.8	1.1	<0.01
Adequate physical activity^e^	45.7	0.9	46.2	1.1	44.9	1.5	0.48
Chronic diseases							
Hypertension	20.4	0.7	22.1	0.9	17.9	1.0	<0.01
Diabetes	5.6	0.4	5.9	0.5	5.3	0.6	0.43
Dyslipidaemia	30.3	0.8	31.7	1.1	28.4	1.3	0.06
Day of dietary survey: weekday	66.4	1.5	67.6	1.6	64.7	1.8	0.09

^a^ Weighted t-tests or χ^2^ tests were used to assess differences between participants who skipped meals and those who did not skip at least one meal a day

^b^ Data are expressed as weighted percentage for categorical variables and as means for continuous variables (with their standard errors).

^c^ Participants who smoked at the time of the survey or had smoked ≥100 cigarettes in their lifetime

^d^ Male and female participants who consumed ≥7 and ≥5 drinks, respectively, at least twice a week.

^e^ Participants who engaged in moderately intense activities for at least 150 minutes during the week, vigorously intense activities for at least 75 minutes during the week, or an equivalent combination of moderately and vigorously intense activities

### Assessment of the meal procurement source according to meal-skipping behaviour

**Fig 2:**
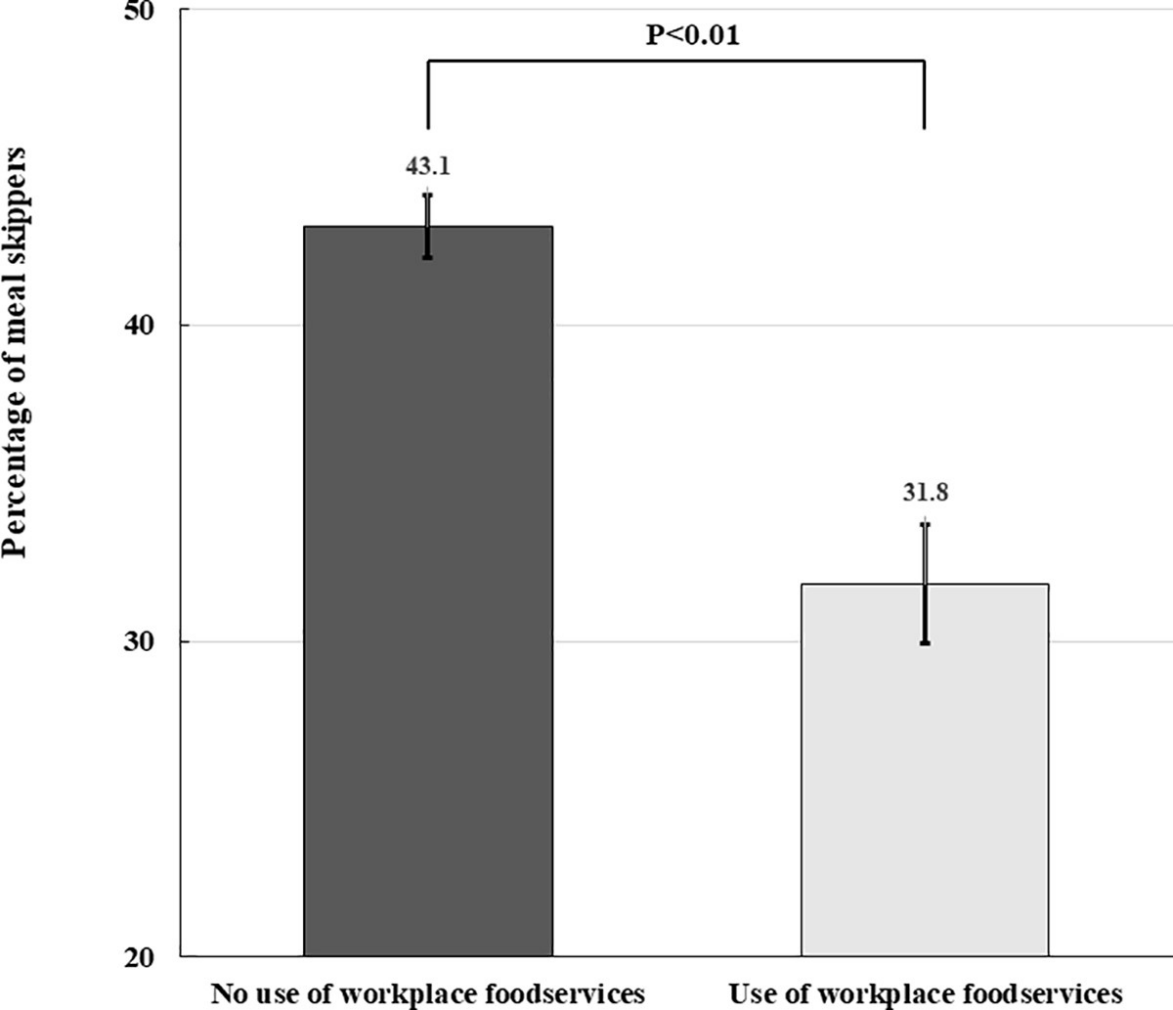
Comparison of the proportion of meal skippers using a weighted χ^2^ test. Of the participants who did not use workplace foodservices and those who used workplace foodservices, 43.1±1.0% and 31.8±1.9% workers skipped at least one meal a day, respectively (P<0.01). There was a significantly high proportion of workers who did not use workplace foodservices in the skipping meal group. Data are presented as weighted percentage with standard errors.

The percentage of workers who skipped meals at least once a day according to the meal procurement source is presented for each meal in Table 2. It was found that the highest percentage of workers skipped their breakfasts (>30%). Among a total of 15,411 meals (three meals a day for 5,137 participants), the rate of meal skipping was 13.6%. Workplace foodservice meals, eating-out meals, and homemade meals accounted for 7.1%, 32.2%, and 47.1% of the total meals, respectively. Of the participants who did not use workplace foodservices and those who used workplace foodservices at least once a day, 43.1±1.0% and 31.8±1.9% workers skipped meals one or more times a day, respectively (P<0.01) (Fig 2). Of the participants who ate homemade meals, the proportion of participants who ate all three meals a day was higher than that of those who skipped meals (74.2 ± 1.6% vs. 31.4 ± 0.9%, P<0.01). However, there was no significant differences in the rate of skipping meals on comparing the eating-out group with the other two groups (P = 0.14).

**Table 2:** Meal groups according to the type of meal and meal-skipping behaviour.

Variables^a^	Total (n = 5,137)		Skipping meals			
			No (n = 3,206)		Yes (n = 1,931)	
	%	SE	%	SE	%	SE
Breakfast						
Skipping meals	31.7	0.9	0	0	76.9	1.1
Workplace foodservice meals	2.6	0.3	3.9	0.4	0.7	0.2
Eating-out meals	12.2	0.5	16.5	0.8	6.0	0.6
Homemade meals	53.6	0.9	79.6	0.9	16.4	1.0
Lunch						
Skipping meals	8.3	0.5	0	0	20.1	1.1
Workplace foodservice meals	15.0	0.7	17.9	0.9	10.9	0.9
Eating-out meals	48.2	0.9	50.0	1.1	45.7	1.3
Homemade meals	28.4	0.8	32.1	1.0	23.2	1.2
Dinner						
Skipping meals	4.8	0.4	0	0	11.6	0.9
Workplace foodservice meals	4.0	0.3	4.5	0.4	3.3	0.5
Eating-out meals	41.7	0.9	37.9	1.1	47.2	1.3
Homemade meals	49.5	0.8	57.6	1.1	37.9	1.2

^a^ Data are expressed as the weighted % and standard errors (SE)

### Association between using workplace foodservices and skipping meals

Table 3 shows the ORs of skipping meals according to the use of workplace foodservices. Skipping meals was significantly associated with not using workplace foodservices, after adjusting for other confounders including sociodemographic, health-related, and meal procurement variables (OR = 3.4; 95% CI = 2.6–4.4; P < 0.01).

**Table 3:** Odds ratios of skipping meals according to the use of workplace foodservices.

	Use of workplace foodservices	No use of workplace foodservices		P-value
	Odds ratio	Odds ratio	95% confidence interval	
Unadjusted	1	1.7	1.4–2.1	<0.01
Model 1^a^	1	3.0	2.3–3.9	<0.01
Model 2 ^b^	1	3.4	2.6–4.4	<0.01
Model 3 ^c^	1	3.4	2.6–4.4	<0.01

^a^ Model 1: adjusted for the place of meal procurement (eating-out and homemade meals)

^b^ Model 2: additionally adjusted for age, sex, household income, marital status, educational level, occupation, smoking status, heavy drinking, adequate physical activity, obesity, chronic diseases (hypertension, diabetes, and dyslipidaemia)

^c^ Model 3: additionally adjusted for snack intake and the day of dietary survey (weekdays/weekend)

## Discussion

In a nationally representative population of Korean workers aged 20–64 years, this study examined the relationship between using workplace foodservices and skipping meals. We found that skipping meals was significantly associated with not using workplace foodservices, after adjusting for potential confounders including sociodemographic and health-related covariates.

There is partial evidence regarding the association between workplace-related factors and skipping meals, with some previous studies showing a potential link between specific working hours or conditions and skipping meals [21–25].

Eating behaviour is known to be affected by a variety of factors including individual perceptions of health or nutritional benefits, physical access, and socioeconomic environmental factors [26,27]. A previous study showed that skipping breakfast was associated with low familial socioeconomic status and sedentary lifestyles [12]. This is partly in line with our findings that workers who skipped meals had lower household incomes. In addition, our finding that workers in the skipping meals group were younger is consistent with that of a recent study in Korea [3]. The association between using workplace foodservices and skipping meals in this study may be potentially explained by factors such as physical access and social environments. Workplace foodservice facilities provide workers with high accessibility and convenience, which likely contribute to the sustainability of their eating behaviours, eventually making them habitual. In addition, the workplace is considered a place where most adults spend a majority of their day, and workplace foodservice facilities are usually located in easily accessible places, which could also contribute toward a reduction in meal-skipping behaviour [28]. Recent studies conducted in the United States and several European countries have suggested that the proximity of food items or availability of eating space where foods can be consumed in the workplace influence workers’ eating behaviours, which is similar to our findings [29,30]. In a qualitative study, some workers reported that access to healthy foods in the workplace is often limited compared with unhealthy foods. However, some other workers reported that their workplace canteen or foodservice provide the opportunity to have proper meals with the provision of a nutritional information and lower cost compared with other food environments outside of the workplace [29,31]. Previous studies have shown the relationship of specific working conditions, such as rotating shift work, temporary employment, and long working hours, with skipping meals [21–25]. These social environmental factors may provide an indirect basis for the effect of using workplace foodservices on meal-skipping behaviour in daily work life, which can have a great impact on maintaining regular schedules, including meal times, while spending the day with other people.

The proportion of meal skippers was lower among users of workplace foodservices than among non-users for every meal, although we could not estimate these statistical significances. Although definitive health benefits of skipping meals or regular meal consumption are unclear, intermittent fasting has emerged recently as an alternative practice to possibly help with obesity and dyslipidaemia; however, there is insufficient evidence of its long-term sustainability in a large sample [24,32–36]. In many countries, irregular eating habits such as meal skipping are not recommended without consultation with doctors, especially in most non-overweight people, due to uncertainty regarding its safety and effectiveness [24,32–36]. Furthermore, there is an association between skipping breakfast and low nutritional adequacy [24]. Therefore, our findings suggest that using workplace foodservices may contribute to having healthy dietary behaviours and high nutritional quality in workers. In addition, given that previous studies have reported a higher rate of meal skipping in temporary workers than in full-time employees [25], the provision of workplace foodservice meals will also help minimise food insecurity in terms of dietary inequality by reducing the incidence of meal skipping.

This study has some limitations. We could not conclude a temporal relationship between using workplace foodservices and skipping meals, as this study had a cross-sectional design. Thus, we could not clearly determine whether the use of workplace foodservices preceded the development of dietary behaviours such as meal skipping. Moreover, despite excluding individuals who were on a diet on the day of the survey to avoid other possible intentional intervention that may influence the results, some participants may have skipped meals because they were not hungry at that time or due to other reasons, and they might not have their meals regardless of their work environment. It is also possible that they are less likely to use workplace foodservices. Therefore, further interventional in-depth studies on this eating behaviour are required in the future. We analysed the data collected using a 24-hour dietary recall method, which may not be appropriate to assess an individual’s habitual diet. However, this limitation is mitigated by the large sample size and the high probability of the identification of diverse dietary behaviours. Further, there is a possibility of response bias, as self-reported data were included in this study. This study was limited to Korean participants; hence, and it may be difficult to translate and generalize the study findings globally.

Despite these limitations, to the best of our knowledge, this is the first study to evaluate the association between using workplace foodservices and skipping meals using recent data collected from a large representative Korean population.

## Conclusions

In conclusion, we found a significant association between using workplace foodservices and skipping meals in Korean adult workers. This study suggests the importance of the provision of workplace foodservices for workers to potentially reduce poor eating behaviours such as skipping meals.
